# Long noncoding RNA Saf and splicing factor 45 increase soluble Fas and resistance to apoptosis

**DOI:** 10.18632/oncotarget.7329

**Published:** 2016-02-11

**Authors:** Olga Villamizar, Christopher B. Chambers, Janice M. Riberdy, Derek A. Persons, Andrew Wilber

**Affiliations:** ^1^ Department of Medical Microbiology, Immunology and Cell Biology, Southern Illinois University School of Medicine, Springfield, Illinois, USA; ^2^ Department of Hematology, St. Jude Children's Research Hospital, Memphis, Tennessee, USA; ^3^ Department of Microbiology, Pontificia Universidad Javeriana, Bogotá, Colombia

**Keywords:** alternative splicing, apoptosis, Fas, long-noncoding RNA, Saf

## Abstract

In multicellular organisms, cell growth and differentiation is controlled in part by programmed cell death or apoptosis. One major apoptotic pathway is triggered by Fas receptor (Fas)-Fas ligand (FasL) interaction. Neoplastic cells are frequently resistant to Fas-mediated apoptosis, evade Fas signals through down regulation of Fas and produce soluble Fas proteins that bind FasL thereby blocking apoptosis. Soluble Fas (sFas) is an alternative splice product of Fas pre-mRNA, commonly created by exclusion of transmembrane spanning sequences encoded within exon 6 (FasΔEx6). Long non-coding RNAs (lncRNAs) interact with other RNAs, DNA, and proteins to regulate gene expression. One lncRNA, Fas-antisense or Saf, was shown to participate in alternative splicing of Fas pre-mRNA through unknown mechanisms. We show that Saf is localized in the nucleus where it interacts with Fas receptor pre-mRNA and human splicing factor 45 (SPF45) to facilitate alternative splicing and exclusion of exon 6. The product is a soluble Fas protein that protects cells against FasL-induced apoptosis. Collectively, these studies reveal a novel mechanism to modulate this critical cell death program by an lncRNA and its protein partner.

## INTRODUCTION

Programmed cell death or apoptosis results from an active cellular response designed to eliminate unnecessary cells and maintain tissue homeostasis. This process is initiated by a variety of stimuli including activation of specific cellular receptors including Fas/Apo-1/CD95, a member of the tumor necrosis factor (TNF) receptor superfamily. Cells expressing Fas undergo apoptosis when treated with agonistic antibodies [1, 2] or upon interaction with Fas ligand (FasL) [3]. Through these receptor/ligand interactions, Fas plays a critical role in cellular proliferation, differentiation, and survival. This delicate balance is affected when soluble forms of this and related surface receptors are produced by alternative splicing of pre-mRNA, a known function of long non-coding RNAs (lncRNAs).

LncRNAs are transcripts longer than 200 nucleotides that have no protein coding potential, but function in an expanding array of cellular processes during normal development and in disease [4-8]. LncRNAs have a relatively low level of evolutionary conservation even within mammalian species [5, 8, 9]. They are generally transcribed at lower levels compared to protein coding genes and can be polyadenylated or not. LncRNAs are found in the nuclear or cytoplasmic compartments and interact with other RNAs, DNA, and proteins either through Watson-Crick base pairing or secondary, stem-loop structures created by RNA folding [8]. These characteristics allow lncRNAs to perform a variety of cellular functions, including to regulate gene expression through transcriptional and post-transcriptional mechanisms [4, 8].

One class of lncRNAs is natural antisense transcripts (NATs). NATs are transcribed from the opposite strand of a protein coding gene and may or may not overlap with portions of coding sequences [10]. It is estimated that approximately two-thirds of human transcripts have at least partial antisense counterparts [11]. Many of these transcripts do not encode proteins, and the majority is expressed at lower levels than the corresponding sense RNA [10, 12-14]. Antisense lncRNAs can control nearly every level of gene regulation through a variety of mechanisms operating in *cis* or *trans* [15]. In *cis*, NATs can interact with the sense transcript or genes within the same region, whereas in *trans*, the interaction is with genes located at distant loci or even other chromosomes [16, 17]. NATs can interact with sense RNA to form RNA duplexes by virtue of their ability to base pair. Consequently, lncRNAs can act as highly specific sensors of pre-mRNA or mRNA, with this interaction resulting in different post-transcriptional outcomes, all of which modulate sense mRNA expression. Through these interactions, lncRNAs have been reported to influence pre-mRNA splicing [18], RNA transport or nuclear retention, or mRNA stability [reviewed in 19].

Alternative splicing regulation by lncRNAs has been shown to occur through chromatin modification [18] and by modulating the levels and subcellular distribution of alternative splicing effector proteins [20]. Fas pre-mRNA can be alternatively spliced to produce sFas, which inhibits Fas-mediated apoptosis [21]. A NAT lncRNA (Fas-AS1 or Saf) transcribed in reverse orientation and from the opposite strand of intron 1 of Fas genomic sequences was shown to modulate Fas pre-mRNA alternative splicing by exclusion of exon 6, which encodes for the Fas transmembrane domain [22]. Saf was found to be expressed at varying levels in different human tissues and in human cancer cell lines and Saf over-expression in Jurkat T cells provided protection from Fas-mediated apoptosis [22]. However, the mechanism by which Saf lncRNA mediates Fas pre-mRNA alternative splicing is unknown.

Here, we provide evidence that Saf interacts with Fas pre-mRNA through complementary base pairing and also interacts with human splicing factor 45 (SPF45) in human cancer cell lines. The interaction of Fas pre-mRNA, Saf, and SPF45 results in skipping of Fas exon 6, increased production of sFas protein, and protection from Fas-mediated cell death. Our results demonstrate that direct interaction between a lncRNA and a splicing factor are sufficient to modulate alternative splicing of a target pre-mRNA. Altogether, these results reveal a novel mechanism to regulate this critical cell death program by an lncRNA and its protein partner.

## RESULTS

### Saf is localized to the nucleus but has limited effects on genome wide transcription

**Figure 1:**
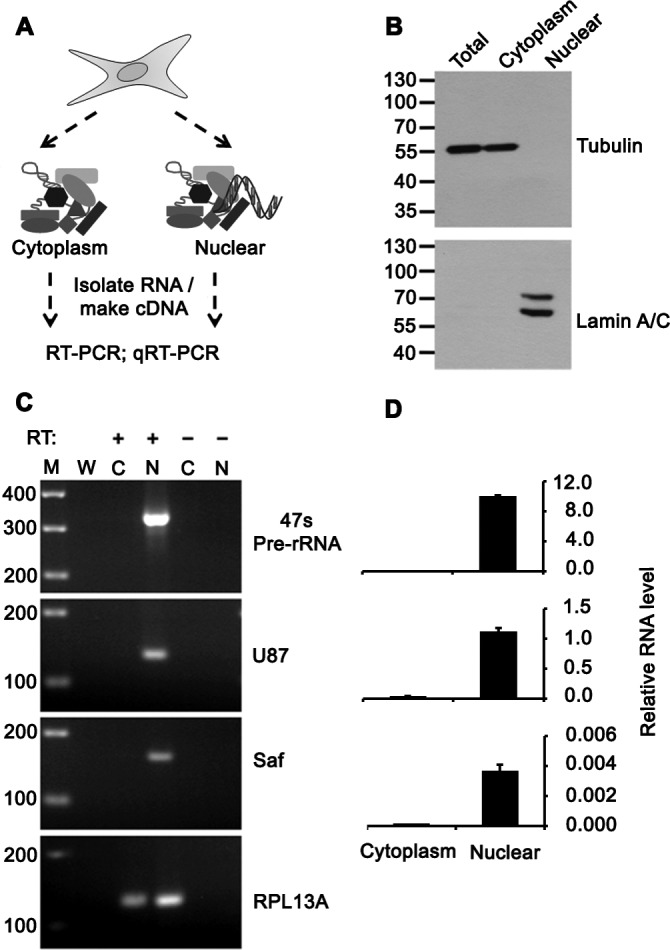
Saf is localized to the nucleus **A.** Schematic diagram showing separation of cells into cytoplasmic and nuclear fractions, RNA isolation, cDNA synthesis, and PCR analyses. **B.** Western immunoblot of total cell and subcellular lysates using antibodies to Tubulin or Lamin A/C to demonstrate fraction purity. Molecular weights (kDal) are indicated. **C.** RT-PCR of 47S pre-rRNA, U87 snoRNA, Saf lncRNA, and RPL13A mRNA for cytoplasm (C) and nuclear (N) fractions with (+) and without (−) reverse transcription (RT). M, 100-bp ladder; W, no template. **D.** Real time quantitative RT-PCR analysis of transcripts detected in indicated fractions relative to RPL13A plotted as mean ± SD of three independent experiments.

**Figure 2:**
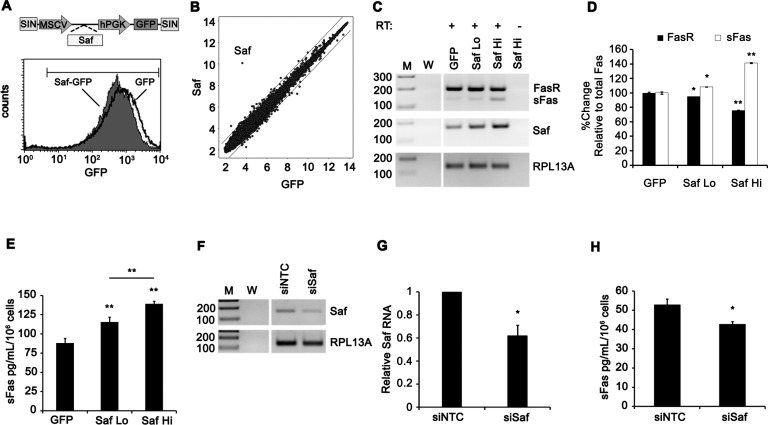
Saf overexpression enhances production of the soluble form of Fas receptor mRNA and protein **A.** Top, diagram of lentiviral vectors encoding GFP or Saf/GFP. Bottom, flow cytometry histogram of GFP expression for transduced cell populations. **B.** Log_2_ normalized intensity of probes plot for microarrays performed on Jurkat T cells. Linear regressions highlight ± 2-fold change where Saf is labeled. **C.** HeLa cells with stable expression of GFP or Saf/GFP were isolated of total RNA and subjected to PCR with (+) and without (−) RT using primers to Saf, exons 5 and 7 of Fas, and the internal control RPL13A. **D.** Densitometry analysis of mRNA products for Fas that include (FasR) or lack (sFas) exon 6 sequences normalized to RPL13A and plotted as percent change relative to total Fas for three independent experiments (GFP control set to 100). **E.** Conditioned supernatants from HeLa cells expressing GFP or Saf/GFP were assayed for soluble Fas (sFas) by ELISA. **F.** siRNA knockdown of Saf in naïve HeLa cells determined by end point RT-PCR and **G.** real time quantitative RT-PCR with normalization to RPL13A (siNTC; set to 1). (H) Conditioned supernatants from these cultures assayed for sFas by ELISA. All quantitative results are plotted as mean ± SD for at least three independent experiments. M, 100-bp ladder; W, no template; NTC, non-targeting control; RT, reverse transcriptase. * indicates *p* < 0.05 (Student's *t*-test); ** indicates *p* ≤ 0.001 (one-way ANOVA with Newman-Keuls post-hoc test). Vertical white lines have been inserted to represent repositioned lanes on the gel images.

Because many lncRNAs are highly compartmentalized [23], we analyzed Saf expression in cytosolic and nuclear fractions of HeLa cells (Figure 1A) and verified fraction purity by western blot (Figure 1B). RT-PCR showed Saf was highly enriched in the nucleus relative to 47S pre-ribosomal RNA and U87 small nucleolar RNA (snoRNA) controls (Figure 1C), representing 97% of total Saf transcripts quantified by real time PCR (Figure 1D). The effect of Saf on genome wide transcription was assessed by transducing Jurkat T cells and K562 cells with lentiviral particles encoding for Saf lncRNA and GFP using independent promoters (see Materials and Methods) or GFP alone (control) (Figure 2A, top) and enriching transduced populations based on expression of GFP by fluorescence-activated cell sorting (FACS) (Figure 2A, bottom). Microarray studies confirmed Saf over-expression in both cell types that was coincident with a 2-fold change in a limited number of probe sets: Jurkat T cells (41 probes; 0.08%, Figure 2B) and K562 cells (21 probes; 0.04%, Supplementary Figure S1). Comparison of genes with altered expression levels revealed that none were conserved between the two cell types or located on the same chromosome as Saf.

### Saf regulates Fas receptor exon 6 splicing and production of soluble Fas

Yan et al. [22] demonstrated that Saf influenced alternative splicing of Fas receptor pre-mRNA to produce a number of shorter transcripts. We tested the ability of Saf to modulate Fas pre-mRNA splicing by engineering HeLa cells to express Saf/GFP or GFP by lentiviral transduction. Cells transduced with Saf/GFP were separated into populations with low or high Saf expression by FACS based on intensity of GFP fluorescence. Alternative splicing of Fas pre-mRNA was monitored by RT-PCR using primers designed to exons 5 and 7 of Fas (Supplementary Table S1B). Saf over-expression significantly enriched for Fas mRNA lacking exon 6 (FasΔEx6), which encodes for a soluble Fas (sFas) protein, compared with GFP control cells (Figure 2C and 2D). ELISA of conditioned supernatants from GFP and Saf transduced cells for sFas protein confirmed that increasing Saf expression generates increasing amounts of sFas protein (Figure 2E; GFP: 88 ± 3; Saf Lo: 116 ± 6; Saf Hi: 139 ± 2 pg/mL/10^6^ cells). Thus, enforced expression of Saf enhances Fas pre-mRNA splicing. Further characterization of the functional effect of Saf on Fas exon 6 alternative splicing was tested by silencing endogenous Saf in HeLa cells using small interfering RNA (siRNA) sequences. Saf specific siRNAs decreased mean Saf levels by 38% (Figure 2F and 2G) relative to non-targeting siRNAs, resulting in a 20% decrease in sFas protein in conditioned supernatants as measured by ELISA (Figure 2H). Collectively, these results demonstrate that Saf regulates Fas exon 6 alternative splicing to increase the production of sFas.

### Saf interaction with Fas pre-mRNA is specific and enriched at splice junction sequences

LncRNAs can interact with other RNAs through complementary base pairing [8, 10]. Several NATs use this mechanism to regulate splicing of overlapping sense transcripts [24]. Saf is encoded within intronic sequences located between exons 1 and 2 of Fas and does not overlap coding sequences. To determine the relative specificity of Saf interaction with Fas RNA, HeLa cell nuclear extracts were treated with proteinase K and resulting cellular RNA mixed with biotin-labeled, *in vitro* transcribed Saf or firefly luciferase (control) RNA (Supplementary Figure S2A). RNA-RNA complexes recovered with magnetic streptavidin beads were converted to cDNA and RT-PCR performed using primers specific for constitutive exons of Fas, four genes with known splice variants (GCIP, HMG2L1, ARHGEF1, and CDK7), and two genes that do not have documented splice products (U87 and RPL13A) (Supplementary Figure S2B). These RNA pull-down experiments revealed that only Saf lncRNA-Fas RNA hybrids were recovered, suggesting the formation of a specific double-stranded RNA intermediate.

**Figure 3:**
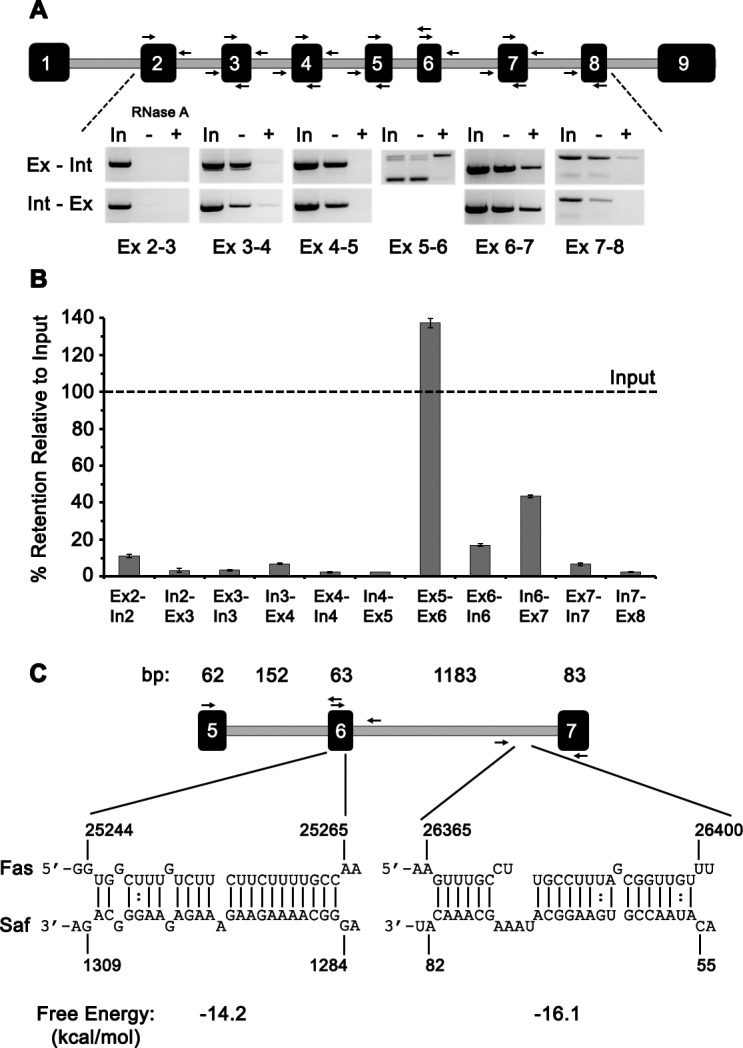
Saf interacts with Fas pre-mRNA at a commonly spliced region **A.** Top, Diagram of Fas pre-mRNA showing the nine coding exons (filled rectangles) and intervening introns (grey lines) where variations in length are used to demonstrate relative size. Arrows indicate the relative position and direction of primers. Bottom, inverse agarose gel images of RT-PCR products generated using primer combinations that amplify exon (Ex) to intron (Int) or Int to Ex sequences for RNA used as input (In) in RNA pulldown experiments or subsequently recovered and left untreated (−) or RNase-A treated (+). **B.** Densitometry analysis of Fas pre-mRNA products for each of the indicated primer pairs reported as percent retained relative to input. Data are plotted as mean ± SD of three independent RNA pulldown experiments. **C.** Top, Enlarged diagram of exons 5 to 7 of Fas pre-mRNA with size (base pairs, bp) of each exon and intron indicated and primers depicted by arrows. Bottom, sequence information for the predicted interaction sites between Fas and Saf and calculated free energy values.

To explore this possibility, RNA pull-down experiments were repeated using biotin-labeled Saf RNA and recovered RNA samples were divided such that one sample was treated with RNAse A before preparing cDNA, while the other sample was used to directly prepare cDNA. Semi-quantitative RT-PCR was performed using primers specific to Fas exon:intron sequences (Figure 3A and Supplementary Table S1C). Amplified products were quantified by densitometry analysis and calculated as percent of input. This RNAse A protection assay revealed the strongest interaction between Saf lncRNA and Fas pre-mRNA occurred at exon 5-6 and exon 6-7 junctions with mean recoveries of 137% and 44% relative to input, respectively; recovery was limited (<15%) for all other regions examined (Figure 3B). To identify potential regions of Saf that interacted with sequences encoded from exon 5 to exon 7 of Fas pre-mRNA we used IntaRNA to predict target sites [25, 26]. This analysis indentified two regions with favorable interaction kinetics (Figure 3C): the first in exon 6 (−14.2 kcal/mol) and second in the intron between exons 6 and 7 (−16.1 kcal/mol). Together, these RNA interaction studies demonstrate a specific association between Saf and Fas pre-mRNA that includes regions within or surrounding the alternatively spliced exon 6.

### Saf interacts with human splicing factor 45 (SPF45)

**Figure 4:**
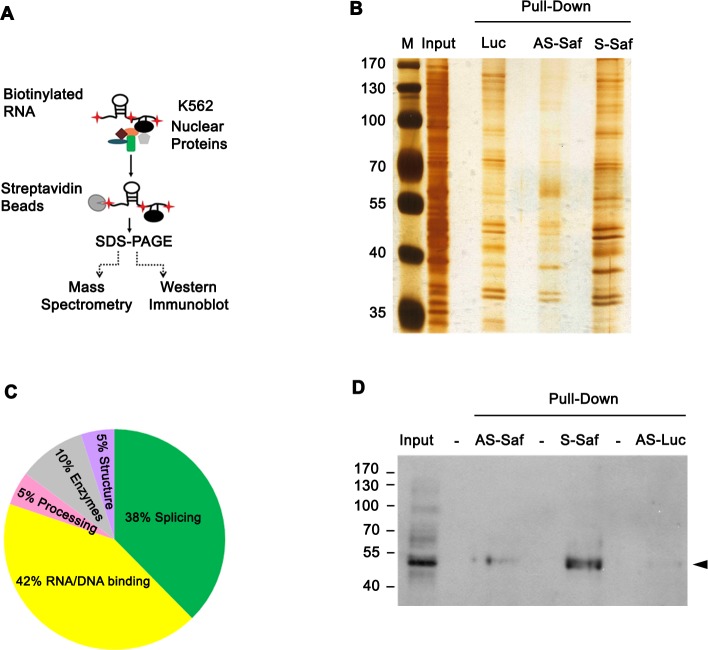
Saf directly interacts with splicing factor SPF45 **A.** Schematic of RNA pulldown experiments using K562 cells. **B.** Silver stained acrylamide gel of total nuclear proteins (input) or nuclear proteins pulled-down with biotin-labeled RNA for firefly luciferase (Luc; control) or Saf lncRNA in anti-sense (AS-Saf) and sense (S-Saf) orientations. M, molecular weight marker (kDal). **C.** Pie chart demonstrating functional clustering of 21 proteins found to interact with Saf by mass spectrometry. **D.** Western blot of nuclear extracts (input) recovered after pulldown of biotin-labeled AS- or S-Saf and reacted with SPF45 antibodies. Molecular weights (kDal) are indicated.

Alternative splicing of pre-mRNA occurs in the nucleus as a function of the spliceosome [27, 28]. To identify nuclear binding partners for Saf, we incubated biotin labeled Saf RNA produced in anti-sense or sense orientations with nuclear protein extracts (Figure 4A). Nuclear lysates and proteins bound to RNA were separated by SDS-PAGE and visualized with silver stain (Figure 4B) or subjected to mass spectrometry for identification. Twenty-one proteins (Supplementary Table S2) were identified from several functional classes (Figure 4C). One of these, human splicing factor 45 (SPF45, also known as RNA-binding motif 17), was reported to have a role in pre-mRNA splicing, and specifically in Fas exon 6 exclusion by a mini-gene assay [29, 30]. The ability of SPF45 to bind Saf was confirmed by repeating the RNA pull-down experiments and performing western blots (Figure 4D).

**Figure 5:**
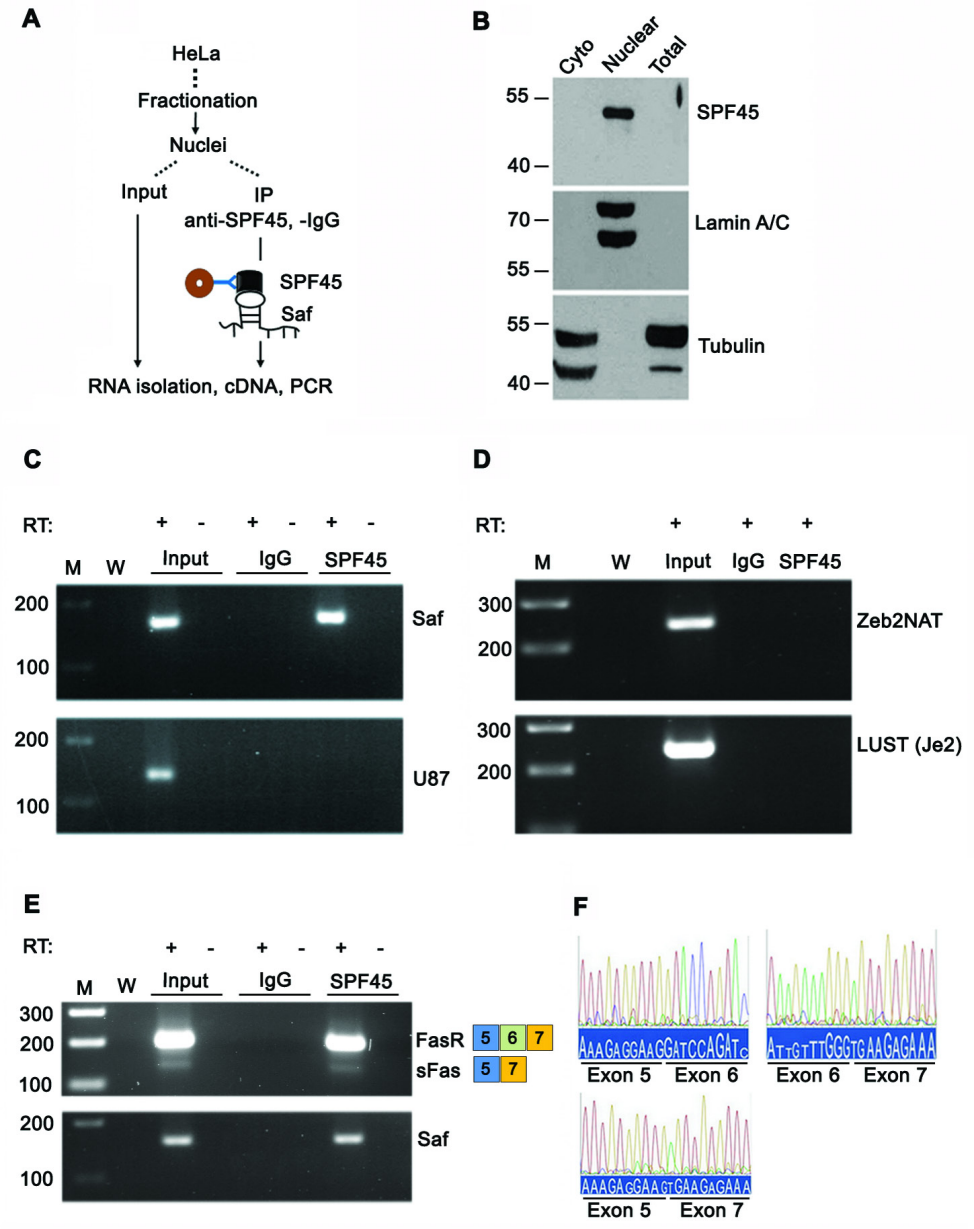
SPF45 interacts with Saf and Fas receptor transcripts **A.** Schema for RNA co-immunoprecipitation experiments performed using nuclear extracts from HeLa cells, which have endogenous expression of Saf. **B.** Western blot of cytoplasmic (cyto), nuclear, or total cell lysates using antibodies to SPF45, Tubulin, or Lamin A/C. Molecular weights (kDal) are indicated. **C.** RNA isolated from nuclear extracts before (Input) and after immunoprecipitation with control IgG or SPF45 antibodies and RT-PCR performed with (+) and without (−) reverse transcription (RT) using primers for Saf, the snoRNA U87, or **D.** two nuclear lncRNAs, Zeb2NAT or LUST (Je2), or **E.** primers to exons 5 and 7 of Fas. M, 100-bp ladder; W, no template. **F.** Chromatograms of sequencing results for amplicons generated for Fas (Exons 5, 6, and 7) and soluble Fas (sFas, Exons 5 and 7) with exon junctions indicated.

Additional confirmation of a specific interaction between Saf and SPF45 was tested by RNA co-immunoprecipitation (RIP) of fractionated cell lysates reacted with antibodies to SPF45 and RT-PCR of recovered RNA (Figure 5A). Western blot demonstrated nuclear localization of SPF45 and verified fraction purity (Figure 5B). Saf lncRNA co-precipitated with SPF45 protein (Figure 5C); however, the small nucleolar RNA U87 (Figure 5C) and two other nuclear lncRNAs also expressed from intronic sequences (LUST and Zeb2NAT; http://www.lncrnadb.org) were not detected (Figure 5D). Therefore, Saf and SPF45 specifically interact, suggesting a functional link to Fas pre-mRNA. This was validated by our ability to detect co-precipitated SPF45 and Fas mRNA with and without exon 6 (Figure 5E), and confirmed through sequence analysis (Figure 5F).

**Figure 6:**
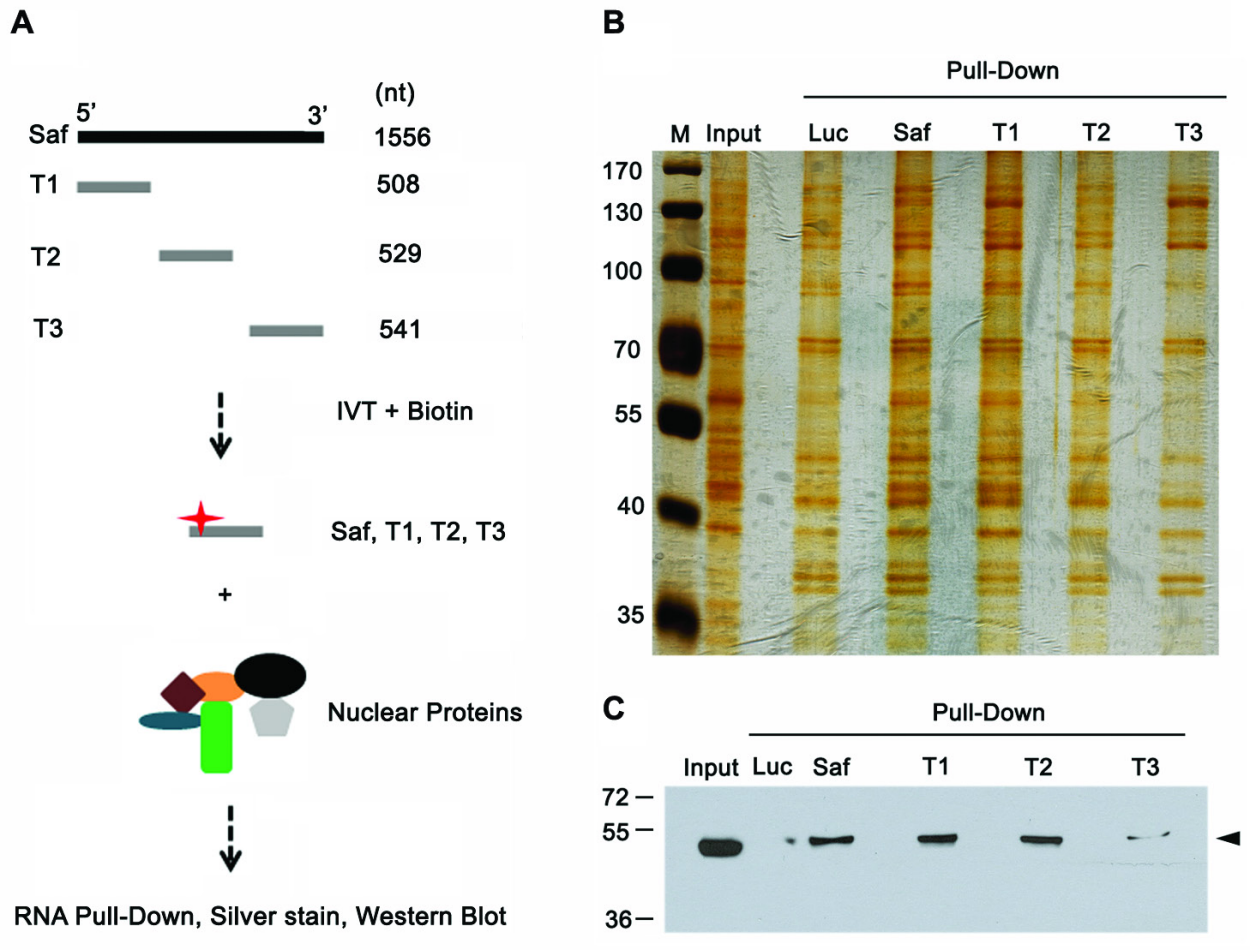
Mapping SPF45-Saf interacting domains **A.** Schematic diagram of RNAs corresponding to different fragments of Saf (T1, T2, or T3) produced by *in vitro* transcription (IVT) in the presence of biotin for RNA pulldown experiments. **B.** Silver stain acrylamide gel of total nuclear proteins before (Input) and after pulldown with biotin-labeled RNAs for firefly luciferase (Luc, control), full-length Saf, or truncated Saf fragments (T1, T2, T3). M, molecular weight marker (kDal). **C.** Western blot of nuclear extracts (input) recovered after pulldown of biotin-labeled Luciferase (Luc), full-length Saf, or truncated Saf transcripts reacted with SPF45 antibodies. Molecular weights (kDal) are indicated.

To further validate the interaction between Saf and SPF45 and determine whether SPF45 interacts with a specific region of Saf, we performed RNA pulldown experiments with truncated versions of Saf (Figure 6A). Nuclear lysates and proteins bound to Saf RNA fragments were separated by SDS-PAGE and visualized with silver stain (Figure 6B) or subjected to western blot for SPF45 (Figure 6C). These assays revealed that the greatest degree of interaction is mediated by sequences located within the first 1037-bp of Saf. Together, the RNA pulldown, RNA immunoprecipitation, and deletion mapping results demonstrate a specific association between SPF45 and Saf.

### SPF45 knockdown reduces Fas splicing and sensitizes cells to Fas-mediated apoptosis

**Figure 7:**
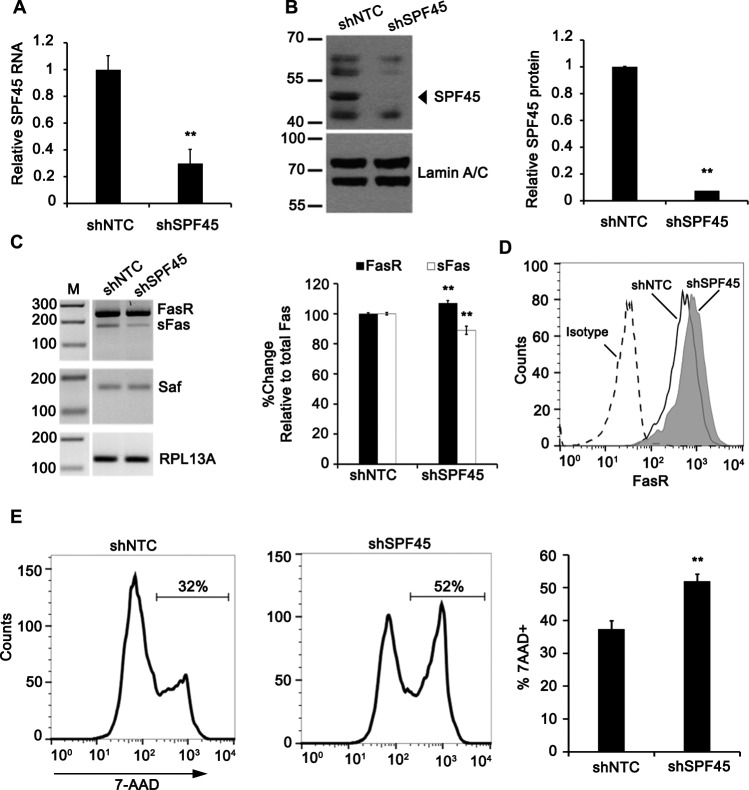
SPF45 knockdown impairs production of soluble Fas and enhances Fas-mediated apoptosis HeLa cells stably expressing shRNA sequences for SPF45 (shSPF45) or a non-targeting control (shNTC) was screened for expression of SPF45 by **A.** Real time quantitative RT-PCR and **B.** Western blot (left), quantitation (right). **C.** Inverse agarose gel images of RT-PCR products from shNTC and shSPF45 cell lines transfected with plasmids encoding a Fas mini-gene (left). M, 100-bp ladder. Densitometry analysis of spliced Fas mRNA products normalized to RPL13A and reported as percent change relative to total Fas (right). **D.** Representative flow cytometry histograms demonstrating surface levels of Fas for shNTC and shSPF45 cell lines reacted with isotype control or anti-Fas antibodies. **E.** Left, representative flow cytometry histograms of 7-AAD incorporation in shNTC and shSPF45 cell lines treated overnight with anti-Fas activating antibody (clone CH11; 100 ng/mL). Right, percentage of 7-AAD positive cells by flow cytometry. Results are plotted as mean + SD for at least three independent experiments. * indicates *p* < 0.05; ** indicates *p* < 0.001 determined by Student's *t*-test.

The functional role of SPF45 on Fas alternative splicing was tested by knocking down SPF45 expression through lentivirus delivery of SPF45 shRNA. SPF45 mRNA levels were determined in cell lines generated from five SPF45 shRNAs and a non-targeting shRNA control (Supplementary Table S3). One shRNA reduced SPF45 transcripts by 69% (Supplementary Table S3 and Figure 7A) and protein (~45 kDa band) by 90% (Figure 7B) and was selected for further study. We hypothesized that loss of SPF45 would have the opposite effect on Fas pre-mRNA splicing as Saf over-expression. This proved correct as evidenced by enrichment for Fas mRNA containing the transmembrane-encoding exon 6 (Figure 7C) and cell surface expression of Fas (Figure 7D; MFI: 1131 ± 234 SPF45 KD *versus* 630 ± 187 control, mean ± SD., *n* = 3, *p* < 0.05). Therefore, SPF45 functions in a similar manner to Saf to regulate Fas exon 6 alternative splicing.

The biological relevance of altered Fas alternative splicing was determined by testing the ability of a Fas agonistic antibody (CH-11) to cause apoptosis of SPF45 knockdown cells. SPF45 knockdown cells demonstrated an increased sensitivity to Fas-mediated signals when compared to non-targeting shRNA control cells (Figure 7E; % 7-AAD positive: 52 ± 2% *versus* 37± 3%, respectively). Therefore, reduction of SPF45 sensitizes cells to Fas signals and leads to increased cell death, supporting the hypothesis that the Saf-SPF45 axis modulates Fas exon 6 alternative splicing.

### Overexpression of Saf in SPF45 knockdown cells does not rescue Fas pre-mRNA splicing

**Figure 8:**
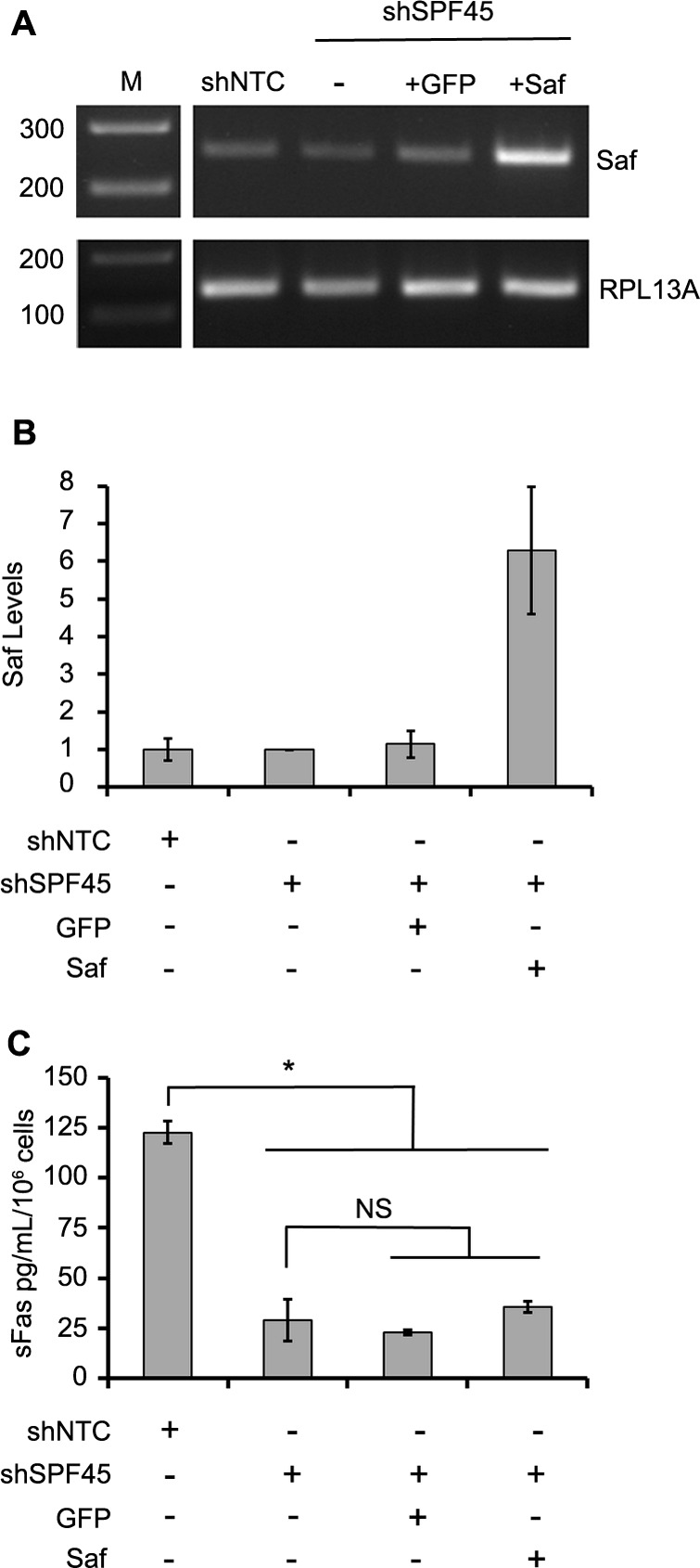
Saf overexpression in SPF45 knockdown cells does not rescue production of soluble Fas **A.** Agarose gel images of Saf transcripts in shNTC stable cells (shNTC; control) and shSPF45 stable cells without transduction (−) or transduced with lentivirus encoding for expression of GPF (+GFP) or Saf/GFP (+Saf) and sorted for GFP expression. **B.** Quantitative real time PCR analysis of Saf levels in the same cell lines, demonstrating an approximately 6-fold increase in Saf expression in the Saf transduced shSPF45 cells. **C.** Conditioned medium for the same cell lines assayed for soluble Fas (sFas) by ELISA. All quantitative results are plotted as mean ± SD. for at least three independent experiments. * indicates *p* < 0.05; NS indicates not significant determined by one way ANOVA with Newman-Keuls post-hoc test. Vertical white lines have been inserted to represent repositioned lanes on the gel images.

To this point, our data demonstrate that independent modulation of Saf or SPF45 influence Fas pre-mRNA splicing, altering the production of Fas proteins (full length Fas or sFas). To verify that Saf and SFP45 interact to stimulate Fas exon 6 skipping, we performed a modified rescue experiment by over-expressing Saf in SPF45 knockdown cells. HeLa cells engineered with SPF45 shRNA were transduced with lentivirus encoding for GFP or Saf/GFP, and the GFP fraction enriched by FACS. Endpoint and quantitative RT-PCR confirmed Saf over-expression (6.3 ± 1.7-fold increase) when compared to cells expressing the non-targeting shRNA, SPF45 knockdown cells not transduced with lentivirus, and SPF45 knockdown cells transduced with the GFP-encoding lentivirus (Figure 8A and 8B). Production of sFas was assayed by ELISA to determine whether over-expression of Saf could rescue the splicing defect induced by SPF45 knockdown. sFas levels were significantly decreased in supernatants collected from SPF45 knockdown cells when compared to non-targeting shRNA controls (Figure 8C; sFas in pg/mL/10^6^ cells: shNTC control 123 ± 6; shSPF45 29 ± 10; *p* < 0.05; mean ± s.d., *n* = 4), confirming our previous results that alternative splicing of Fas was reduced by SPF45 knockdown. However, Saf over-expression in SPF45 knockdown cells had no statistically significant effect on production of sFas when compared to SPF45 knockdown cells that were not transduced (shSPF45 above) or GFP transduced (Figure 8C, shSPF45 KD ± GFP: 23 ± 1; shSPF45 KD ± Saf: 36 ± 3 pg/mL/10^6^ cells, mean ± s.d., *n* = 4). These findings indicate that elevated levels of Saf are not sufficient to enhance Fas splicing when SPF45 is limited, and support the hypothesis that Saf and SPF45 co-participate in modulating Fas pre-mRNA splicing and the production of full length or sFas, thereby altering apoptosis.

## DISCUSSION

RNA is flexible and can assume a variety structures that allow it to interact with other RNAs, DNA and proteins. This unique feature allows RNA to perform a range of important regulatory roles in the cell, including pre-mRNA splicing. LncRNAs are known to function in pre-mRNA splicing through various mechanisms. Examples of these lncRNAs include several natural antisense transcripts (NATs) [31-34], repeat-containing lncRNAs [35], and the intergenic lncRNA lung adenocarcinoma transcript 1 (MALAT1) [20]. Some responsible mechanisms include RNA:RNA duplexing [31, 36] with overlapping sense transcripts and regulation of functional levels of splicing factors [20]. We demonstrated that Saf is localized to the nucleus, but over-expression had limited effects on genome-wide transcription. We found that Saf interacted with Fas pre-mRNA at regions that flank the transmembrane domain (exon 6) and splicing factor SPF45 to regulate production of a sFas protein which protected cells from Fas-mediated apoptosis. Production of sFas was dependent upon expression of both Saf and SPF45.

Alternative splicing is a regulated process that results in the generation of distinct proteins from a single primary transcript [37]. Fas pre-mRNA is alternatively spliced to produce a number of soluble isoforms [38]; the most abundant, well-studied isoform, FasΔEx6, lacks the hydrophobic transmembrane-spanning domain [21, 39]. This soluble version of Fas can bind FasL in the extracellular space and impair normal signaling [21, 39]. Dysregulation of the Fas signaling pathway is seen in numerous pathological conditions, including cancer, neurological, cardiovascular, infectious/viral, autoimmune and hematopoietic disease. We show that Saf interacts with Fas pre-mRNA at regions that flank exon 6, and that Saf levels influence production of sFas. These effects could cause abnormal Fas signaling and pathology in any of the aforementioned conditions.

LncRNAs have been identified to either promote [40-42] or inhibit [5, 43] apoptosis, through mechanisms that include alternative splicing of a pre-mRNA. Furthermore, alternatively spliced gene products are known to play important roles in promoting or preventing cell death [44]. Regulation of Fas alternative splicing has been demonstrated through proteins that promote exclusion of exon 6 [45]. One of these proteins is SPF45, a component of splicing complexes. Expressed at low levels in most tissues, SPF45 functions in the recognition and activation of the 3′ splice site AG within introns [29]. SPF45 over-expression is seen in breast, ovarian, bladder, colon, lung, pancreatic, and prostate cancer with proposed roles in proliferation and chemotherapy resistance [46] as well as cell migration and invasion [47]. We show that SPF45 directly interacts with Saf to promote alternative splicing of Fas. Furthermore, knockdown of SPF45 decreased alternative splicing of Fas pre-mRNA translating to reduced sFas production, accumulation of Fas on the cell surface, and increased sensitivity to Fas-dependent apoptosis. While the role of SFP45 in Fas exon 6 alternative splicing is well characterized, SPF45 is also involved in alternative splicing of many other pre-mRNAs. The mechanism by which a single splicing factor is targeted to a specific pre-mRNA and exon/intron border is not well known, but we propose that lncRNA Saf acts to recruit SPF45 to Fas pre-mRNA, specifically to the exon 6 region, to promote exon 6 exclusion. Regulation of this complex may depend upon post-translational modification of SPF45 where phosphorylation has been shown to regulate its role in alternative splicing of Fas [30, 47]. As these are the first studies describing a role for a lncRNA in SPF45-mediated alternative splicing, our results suggest that lncRNAs with similar function are yet to be discovered. Sequence analysis of RNA immunoprecipitated with SPF45 or other splicing factors could identify lncRNAs with complementary function.

**Figure 9:**
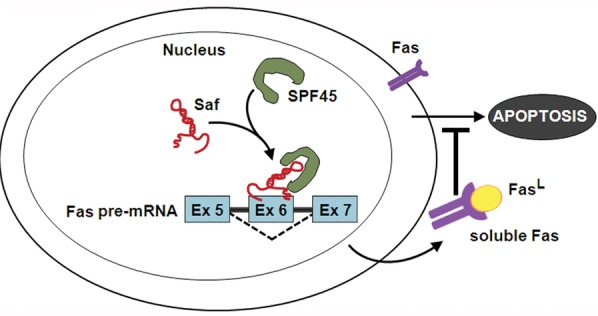
Model of Saf and SPF45 in attenuating Fas-mediated apoptosis LncRNA Saf guides SPF45 to Fas receptor (Fas) pre-mRNA, specifically to the exon 6 (Ex 6) region, facilitating removal of transmembrane sequences by alternative splicing. The result is diminution of Fas on the cell surface and increased production of soluble Fas that sequesters Fas ligand (Fas^L^) and renders cells less sensitive to Fas-mediated apoptosis.

From our collective data, we propose a model where Saf interacts with Fas pre-mRNA to guide SPF45 to this unprocessed transcript and direct alternative splicing of Fas exon 6 (Figure 9). The result is increased production of sFas that protects cells against Fas-mediated apoptosis. This mechanism can be generalized to a wide-variety of cell types functioning in normal or disease conditions to include control of erythropoiesis [48], depletion of antigen-activated T cells [49], development of acute leukemia [50], survival of leukemic large granular lymphocytes [51], and autoimmune lymphoproliferative disorder (ALPS) [52], where increased production of sFas has been suggested to block apoptosis. Further characterization of the interaction between Saf, SPF45, and Fas pre-mRNA may reveal additional levels of complexity and present novel strategies aimed at re-establishing proper Fas signaling in a variety of pathological conditions.

## MATERIALS AND METHODS

### Vector construction

*Saf lncRNA lentivirus.* Total RNA (350ng) isolated from bone marrow CD34^+^ cells was reverse transcribed (High Capacity RT kit; Applied Biosystems) with a primer corresponding to the 3′-end of Saf (Supplementary Table S1A). cDNA (1μg) was PCR amplified using Saf lncRNA primers (Supplementary Table S1A) and Phusion Hi-Fidelity Taq polymerase (Fermentas). A 1536-bp amplicon representing the full length Saf lncRNA was gel extracted (QIAquick, Qiagen), A-tailed and inserted into pCR2.1 TOPO/TA (Invitrogen) to create pCR2.1/Saf and sequence verified (GenScript). *Tth*111I (5′) and *Age*I (3′) restriction sites were introduced by PCR (primers: Supplementary Table S1A) and a *Tth*111I to *Age*I fragment subcloned between the murine stem cell virus (MSCV) and human phosphoglycerate kinase (hPGK) promoters in pCL20c/MSCV-hPGK-GFP lentiviral vector. The resulting vector directs expression of lncRNA Saf under control of the MSCV promoter and independently expresses GFP under control of the hPGK promoter and was used to prepare lentiviral particles.

*Saf lncRNA in vitro transcription.* The T3 promoter binding sequence (5′) and *Not*I restriction site (3′) were introduced by PCR (primers: Supplementary Table S1A) into pCR2.1/Saf to create pCR2.1/Saf *in vitro* and used for Saf *in vitro* transcription.

### Cell culture and transfection

Human embryonic kidney (HEK) 293T, erythroleukemia K562, Jurkat T cells, and HeLa cervical carcinoma cells were cultured in DMEM (Mediatech) supplemented with 10% (v/v) fetal bovine serum (FBS; HyClone laboratories) and antibiotics. Plasmids were transfected using Lipofectamine 2000 (Invitrogen) and analyzed 24 hours later.

### Western immunoblot

Whole cell lysates, prepared with M-PER plus HALT protease inhibitors (Thermo Scientific), were separated by SDS-PAGE and transferred onto PVDF membranes (Immobilon-P, Millipore). Primary antibodies recognizing SPF45 were from Santa Cruz Biotechnology; GAPDH antibodies were from Sigma; Lamin A/C and α-tubulin were from Active Motif. HRP-conjugated secondary antibodies were from Thermo Scientific.

### Subcellular fractionation

Cells were lysed with a buffer containing 50mM KCl, 25mM HEPES (pH 7.8), 1mM phenylmethylsulfonyl fluoride (PMSF), 10 μg/mL leupetin, 25 μg/mL aprotinin, 100 μM dithiothreitol (DTT) and 0.5% NP-40, and the resulting lysates centrifuged at 2,700 × *g* for 5 min at 4°C. The supernatant was used as the cytosolic fraction. Pelleted material was washed, incubated in nuclear extraction buffer (500mM KCl, 1.5mM MgCl_2_, 25mM HEPES, 1mM PMSF, 10 μg/mL leupeptin, 25 μg/mL aprotonin, 100 μM DTT and 10% glycerol) on ice for 30 min and the nuclear fraction collected by centrifugation at 20,000 × *g* for 10 min at 4°C.

### Saf lncRNA subcellular localization

Cells were separated into cytoplasmic and nuclear fractions and RNA (50ng) isolated from each fraction reverse transcribed into cDNA as described above. End point PCR (30 cycles) was performed using cDNA (100ng), PCR Master Mix (Thermo Scientific), and primers to 47S pre-rRNA, U87, Saf, or RPL13A (Supplementary Table S1B). Amplified products were resolved on 3% TAE-agarose gel containing ethidium bromide and photographed. Quantitative PCR (qPCR) was performed on a StepOne Plus thermocycler (Applied Biosystems) using cDNA (100 ng), iTaq Universal SYBR Green Super Mix (BioRad), and primers to 47S pre-rRNA, U87, or Saf, and normalized to RPL13A (Supplementary Table S1B).

### Saf lncRNA *in vitro* transcription

Sense (T3) and anti-sense (T7) biotinylated (Bio-16-UTP, Life Technologies) Saf lncRNA transcripts were generated from pCR2.1/Saf *in vitro* (MegaScript, Ambion). Unlabeled Saf RNA was prepared in the absence of bio-16-UTP. RNA was precipitated with lithium chloride and stored at −80°C.

### RNAse A protection assay

HeLa cells (20×10^6^) were fractionated as described above and nuclear extracts treated with Proteinase K (0.1 μg) for 90 minutes at 42°C before being isolated of total RNA (Purelink RNA mini kit, Ambion). An aliquot of RNA was stored at −20°C to be used as input control in the PCR analysis. RNA was mixed with yeast tRNA (Sigma) pre-blocked magnetic streptavidin beads (Invitrogen) and biotin-labeled Saf (8 μg). Reactions were incubated overnight at 4°C with end-over-end rotation. The following day, samples were washed (100 mM HEPES pH 7.8, 500mM LiCl, 10mM EDTA, and 10 mM DTT). RNA was separated into two tubes and one sample treated with RNAse A (7 units; ActiveMotif) for 30 minutes at 37°C to digest single-stranded RNA. Samples were heat inactivated (95°C for 10 minutes) and cDNA prepared as described above. The cDNAs (1 μg) were PCR amplified for 40 cycles using PCR Master Mix (Thermo Scientific) and primers designed to recognize exon and intron sequences of Fas pre-mRNA with products ranging from ~275-bp to 660-bp (Supplementary Table S1C). Amplified products were resolved on 3% TAE-agarose gel containing ethidium bromide and photographed.

### RNA pull-down, silver stain and mass spectrometry

RNA pull-down was performed according to [53]. Nuclear extracts were obtained from K562 cells as described above. Extracts (~300 μg) were mixed with denatured RNA (8 μg) corresponding to unlabeled Saf (sense) or biotinylated anti-sense or sense Saf and the mixtures incubated overnight at 4°C with yeast tRNA (Sigma) pre-blocked streptavidin beads (Invitrogen). Beads were collected by centrifugation at 20,000 × g for 1 min at 4°C and protein samples separated by SDS-PAGE. Resolved proteins were visualized using silver stain (Pierce).

For mass spectrometry, immunoprecipitated proteins were separated by SDS-PAGE, gel pieces digested with trypsin and peptides injected with the assistance of Genomic Solutions robotic system. Peptides were eluted and identified using a tandem time-of-flight mass spectrometer (4700 Proteomics Analyzer; Applied Biosystems). The raw data was searched in Mascot, SEQUEST or Protein Prospector against the human subset of the SwissProt database. Saf-protein interactions were confirmed by repeating the RNA-pulldown experiments and immunoprecipitated complexes verified by western blot.

### RNA co-immunoprecipitation (RIP)

Immunoprecipitation of endogenous protein-RNA complexes was performed using HeLa cell nuclear extracts (described above). Extracts were incubated with anti-SPF45 and protein A/G magnetic beads (Thermo Scientific) overnight at 4°C. RNA was isolated from the precipitated complexes and reverse transcribed (Life Technologies). RT-PCR was performed using cDNA (1 μg), PCR Master Mix (Thermo Scientific), and primers to Saf, U87, Zeb2NAT, Lust, or Fas (Supplementary Table S1B). Amplified products were resolved on 3% TAE-agarose gel, stained with ethidium bromide and photographed.

### Saf lncRNA overexpression

Lentiviral vector particles pseudotyped with vesicular stomatitis virus G protein (VSV-G) were prepared for pCL20c/Saf-ires-GFP or pCL20c/ires-GFP by transient transfection of HEK293T cells using the calcium phosphate precipitation technique [54]. Vector preparations were titered on K562 cells based on GFP expression as determined by flow cytometry. Cells were transduced at multiplicity of infection (m.o.i.) of 1, 3, or 10 in the presence of polybrene (8 μg/μL; Sigma). Transduced cell populations were enriched by FACS (FACSAria, BD Biosciences) based on GFP expression after 5-7 days. For select experiments, cells were further separated into GFP-low and GFP-high populations by FACS. Saf levels were determined by end-point RT-PCR to verify coordinate expression patterns between GFP and Saf in these populations.

### Microarray analysis

Total RNA was isolated from FACS enriched GFP (control) and Saf/GFP lentiviral transduced Jurkat T cells and K562 cells. RNA (100 ng) was reverse transcribed into cDNA using 3′ IVT Express kit (Affymetrix) and hybridized to an HG-U133_Plus2 cartridge (Affymetrix) permitting analysis of over 47,000 transcripts. Gene expression values were calculated by robust means analysis (RMA) model with simple fold-change applied to identify differential expression and the log_2_ normalized intensity of filtered probes plotted for each cell type. All microarray data have been deposited in the Gene Expression Omnibus (http://www.ncbi.nlm.nih.gov/geo/).

### Saf lncRNA knockdown by siRNA

A pool of four siRNAs specific for Saf or non-targeting siRNAs (Lincode siRNA, Thermo Scientific) were transfected into HeLa cells at 75 nM using DharmaFECT 1 (Thermo Scientific). Cells were incubated for 48 hours then RNA was isolated using PureLink RNA mini kit (Invitrogen) and reverse transcribed into cDNA (VILO; Life Technologies). RT-PCR was carried out using cDNA (100 ng), PCR Master Mix (Thermo Scientific), and primers to Saf or RPL13A (Supplementary Table S1B). Amplified products were resolved on 3% TAE-agarose gel stained with ethidium bromide and photographed. Quantitative PCR (qPCR) was performed as described above for Saf and normalized to RPL13A.

### SPF45 shRNA knockdown

Five lentiviral vector constructs encoding for expression of shRNA sequences specific to SPF45 (Supplementary Table S3) were purchased from The RNAi Consortium (Sigma). Control shRNAs specific to placenta growth factor (Sigma) were obtained through Dr. Donald Torry (SIU School of Medicine). Lentivirus particles were generated and HeLa cells transduced as described above. Stable cell lines were selected with puromycin (0.5 μg/mL); pools of puromycin-resistant cells were evaluated by qPCR and western blot for SPF45 mRNA and protein levels, respectively, compared to non-targeting control shRNA expressing cells. From these studies, clone TRCN0000231429 was selected for use in the knockdown studies (Supplementary Table S3).

### Quantification of end-point PCR products

Agarose gels were photographed with a BioDoc-It 220 Imaging System equipped with a 1.3 megapixel camera (UVP, Analytik Jena) and images acquired as TIFFs. Images were inverted (Adobe Photoshop) and the number of pixels in each band measured using ImageJ.

### Flow cytometry analysis

Flow cytometry studies were performed on FACSCalibur or FACSAriaII (BD Biosciences) using CellQuest v5.2.1 or FlowJo v10.0 analysis software to monitor the following:

*Fas receptor.* HeLa cells were reacted with APC-conjugated mouse anti-human CD95 (DX2, BD Biosciences); mouse anti-human IgG-APC was used as an isotype control. The geometric mean of fluorescence intensity (MFI) was determined for each condition.

*Apoptosis.* HeLa cells were treated overnight with 100 ng anti-Fas activating antibody (CH-11, Millipore), washed with PBS, incubated with 7-amino-actinomycin D (7-AAD, Millipore) for five minutes and the percent 7-AAD positive cells determined.

### Soluble Fas (sFas) ELISA

Conditioned supernatants were collected from HeLa cells 48 to 72 hours after plating, and used immediately or stored at −20°C. Adherent cells were harvested with trypsin and viable counts performed by trypan blue exclusion. Supernatants were assayed for sFas by ELISA (R&D Systems) according to the manufacturer's protocol and optical density measured at 450nm using a Multiskan Spectrum ELISA reader (Thermo Scientific). Levels of sFas are expressed as pg/mL/10^6^ viable cells.

### Statistical analysis

Microsoft Excel or Prism 5 (GraphPad) was used to determine descriptive statistics (mean ± SD) and significant differences between mean values determined by unpaired Student's *t*-test (two-tailed) or one-way ANOVA with Newman-Keuls post-hoc test. *P-values* are indicated by asterisks in the figures with level of significance reported.

## SUPPLEMENTARY MATERIAL FIGURES AND TABLES


