# Supplementary material for: Long noncoding RNA Saf and splicing factor 45 increase soluble Fas and resistance to apoptosis
Source: Oncotarget. 2016 Feb 11;7(12):13810–26. doi: 10.18632/oncotarget.7329 (PMC4924680; doi:10.18632/oncotarget.7329)
Supplement: Supplementary file 1 [file oncotarget-07-13810-s001.pdf]

Long noncoding RNA Saf and splicing factor 45 increase soluble Fas and resistance to apoptosis

Supplementary Material

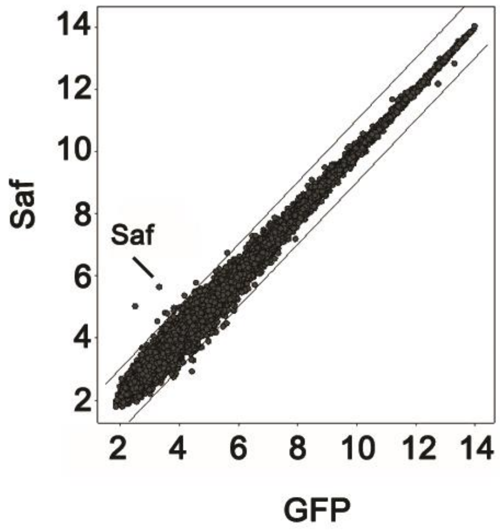

Figure S1. (Related to Figure 2) Whole genome array of K652 cells with Saf overexpression. Log_2 normalized intensity of probes from Affymetrix HG-U133_Plus2 arrays performed on K562 cells. Linear regressions highlight $\pm$ 2-fold change in transcript levels where the dot corresponding to Saf is labeled.

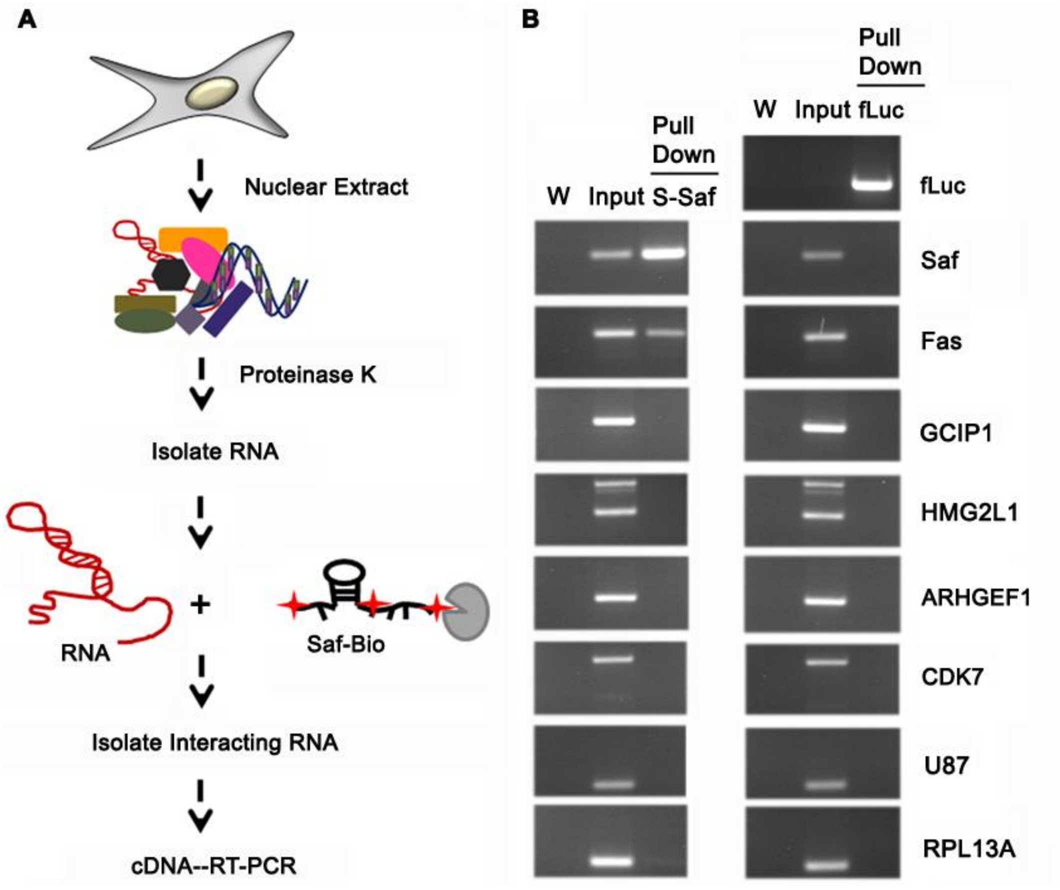

Figure S2. (Related to Figure 3) Saf interacts with Fas mRNA. (A) Schematic diagram of the RNA pull-down assay. (**B**) Agarose gel images of RT-PCR products generated for the indicated genes from RNA before (input) and after RNA pull-down using biotin-labeled versions of Saf (Left) or firefly luciferase (fLuc, Right). W, no template control.

Table S1. Primers used in these studies.

A. Primers used for PCR cloning

Name	Forward	Reverse
Saf lncRNA	TTCCAAGTAATTAGCACTTTGC	ACGTAGGAAATAAGTCAGC
Tth111I-Saf-AgeI	GACTGCGTCTTCCAAGTAATTAGCACTTTGC	ACCGGTACGTAGGAAATAAGTCAGC
T3-Saf-NotI	ATTAACCCTCACTAAAGTTCCAAGTAATTAGC ACTTTGC	GCGGCCGCACGTAGGAAATAAGTCAGC

B. Primers used for RT-PCR and qRT-PCR

Primer Name	Forward	Reverse
Saf	ATCAGCGAACAGCCTGAGTT	GAGCCATGTAGTGGGGAAGA
47S pre rRNA	GCTGACACGCTGTCCTCTGG	GAGAACGCCTGACACGCACG
snoRNA U87	ATGGGATCATGGAGCAGCTG	TCACACCCATGACTGCCACT
RPL13A	CCTGGAGGAGAAGAGGAAAGAGA	TTGAGGACCTCTGTGTATTTGTCAA
Fas (endogenous)	ATGTGAACATGGAATCATCAAGG	GGAGATTCATGAGAACCTTGG
LUST	AACAAGCCGCCTGAACTAAA	CTGATTCCCCCAGTCTTCAA
Zeb2NAT	GTCAAGCCTTTGGCATCATT	GGGCAGAGAACTTTGTTCCA

C. Primers used for RNAse A protection assay

Primer Name	Sequence	Pair	Product Size (bp)
Ex2 For	TGCCCAAGTGACTGACATCA	Ex2 For + In2 Rev	660
In2 Rev	CGTCTAGTTATTCAGCTGC		
In2 For	CACTGTTTCTTATCAGGATTTG	In2 For + Ex3 Rev	553
Ex3 Rev	TGGGCTTTGTCTGTGTACTCC		
Ex3 For	CTAGGGACTGCACAGTCAATG	Ex3 For + In3 Rev	490
In3 Rev	CTGTGTCCTTGTATCTGCAG		
In3 For	CATCAGAGTCTCTGAGTGAG	In3 For + Ex4 Rev	495
Ex4 Rev	CAAGGGTCACAGTGTTCAC		
Ex4 For	GGACCCAGAATACCAAGTGC	Ex4 For + In4 Rev	475
In4 Rev	GACACATTCATGTCTTCACCTCC		
In4 For	CATGAACCTCTTGAGTCTCC	In4 For + Ex5 Rev	562
Ex5 Rev	CTCTTTGCACTTGGTGTTGC		
Ex5 For	ATGTGAACATGGAATCATCAAGG	Ex5 For + Ex6 Rev	276
Ex6 Rev	CCAAACAATTAGTGGAATTGGC		
Ex6 For	CCAGATCTAACTTGGGGTGG	Ex6 For + In6 Rev	419
In6 Rev	GCAATGTGTTTCCTGGGAATGCC		
In6 For	GGACAGAGAGTGGTGCTTGCC	In6 For + Ex7 Rev	354
Ex7 Rev	GGAGATTCATGAGAACCTTGG		
Ex7 For	GGAAGTACAGAAAACATGC	Ex7 For + In7 Rev	516
In7 Rev	CCTATGCACCCACAAACACAAGC		
In7 For	CCAGGGCTCAGTGGGAGTTAGG	In7 For + Ex8 Rev	454
Ex8 Rev	CAGATAAATTTATTGCCACTG		

Table S2. (Related to Figure 4) Top twenty-one Saf interacting nuclear proteins identified by mass spectrometry. Two biological replicates using mass-spectrometry analysis. *confirmed by RNA-coimmunoprecipitation and western blot with specific antibodies.

Name	Description	Accesion No.	General Characteristic
SSB	Sjogren Syndrome Antigen B*	P05455	RNA/DNA binding
NRF	NF-kappaB repressing factor	A3F769	RNA/DNA binding
SKIV2L2	ATP-dependent helicase SKIV2L2	P42285	Enzyme
SPF45	Splicing Factor 45*	Q5W009	pre-mRNA splicing
COIL	Coilin	P38432	RNA/DNA binding
ZCCHC8	Zinc finger CCHC domain-containing protein 8	Q6NZY4	pre-mRNA splicing
PHF5A	Splicing factor 3B-associated 14 kDa protein	Q7RTV0	pre-mRNA splicing
SF3A	Splicing factor 3A	Q15459	pre-mRNA splicing
DDX18	ATP-dependent RNA helicase DDX18	Q9NVP1	RNA/DNA binding
H3F3A	Histone H3-3 A subunit	B4DEB1	Structure
U2AF1	Splicing factor U2AF 35 kDa subunit	Q01081	pre-mRNA splicing
PRKRA	Interferon-induced protein kinase	O75569	Enzyme
CPSF1	Cleavage and polyadenylation specific factor 1	Q10570	RNA/DNA binding
PDCD11	Programmed Cell Death Protein 11 RRP5	Q14690	pre-mRNA splicing
SNRPF	Small nuclear ribonucleoprotein F	P62306	pre-mRNA splicing
SNRPE	Small nuclear ribonucleoprotein E	P62304	pre-mRNA splicing
RCC2	Regulator Of Chromosome Condensation protein 2	Q9P258	RNA/DNA binding
MSH2	DNA mismatch repair protein	E9PHA6	RNA/DNA binding
ZC3HAV1	Zinc finger CCCH-type antiviral protein 1	C9J6P4	RNA/DNA binding
GAR1	H/ACA ribonucleoprotein complex subunit 1	Q9NY12	RNA/DNA binding
XRN2	5'-3' exoribonuclease 2	B4DZC3	RNA processing

Table S3. (Related to Figure 7 and Experimental Procedures) RNAi Consortium shRNA clones used in this study. Knockdown (KD) efficiency determined by qRT-PCR normalized to RNaseP and expressed relatve to PGF (NTC) control. NTC, non-targeting control.

**Clone Number**	**Target Gene**	**Target Sequence**	**KD Efficiency (%)**
TRCN0000058238	PGF (NTC)	TGCAGCTCCTAAAGATCCGTT	Control
TRCN0000001201	SPF45	GATGAAGCAGTACGGATATTT	0
TRCN0000001203	SPF45	AGATGAAGATTATGAGCGAGA	27
TRCN0000010600	SPF45	CCGGTGATCCTTAAATGAACT	63
TRCN0000231429	SPF45	ATACTTAAGTGTCCTACTAAA	69
TRCN0000231430	SPF45	GATGAAGCAGTACGGATATTT	33
